# Prognostic value of admission ROTEM in trauma: enhancing 30-day all-cause mortality prediction using machine learning

**DOI:** 10.1007/s00068-025-02959-8

**Published:** 2025-10-21

**Authors:** Villiam V. Kildal, Martin Dahlberg, Carl Henrik Ek, Anders Oldner, Agneta Wikman, Carl Magnus Wahlgren, Mattias Günther

**Affiliations:** 1 Department of Clinical Science and Education, Section of Anesthesiology and Intensive Care, Karolinska Institutet, Södersjukhuset, Stockholm, Sweden; 2 Department of Perioperative Care and Intensive Care, Södersjukhuset, Stockholm, Sweden; 3 Department of Surgery, Södersjukhuset, Stockholm, Sweden; 4 Department of Computer Science and Technology, University of Cambridge, Cambridge, UK; 5 Department of Physiology and Pharmacology, Section of Anaesthesiology and Intensive Care, Karolinska Institutet, Stockholm, Sweden; 6 Department of Perioperative Medicine and Intensive Care, Karolinska University Hospital, Stockholm, Sweden; 7 Department of Medicine, Karolinska Institutet, Huddinge, Stockholm, Sweden; 8 Department of Clinical Immunology and Transfusion Medicine, Karolinska University Hospital, Stockholm, Sweden; 9 Department of Molecular Medicine and Surgery, Karolinska Institutet, Stockholm, Sweden; 10 Department of Vascular Surgery, Karolinska University Hospital, Stockholm, Sweden; 11 Department of Neuroscience, Section of Experimental Traumatology, Karolinska Institutet, Stockholm, Sweden

**Keywords:** Thromboelastometry, Thromboelastographynull, Viscoelastic hemostatic assays, Coagulation, Trauma, Mortality, Outcome, Prediction, Machine learning, Artificial intelligence, Intensive care

## Abstract

**Background:**

Haemorrhage is a leading cause of trauma death, yet early coagulation markers are rarely used to predict long-term outcomes. This study assessed whether a single admission rotational thromboelastometry (ROTEM) test could independently predict 30-day all-cause mortality and improve existing trauma scores.

**Methods:**

We conducted a retrospective cohort study of 1,498 adult trauma patients admitted to a Level 1 trauma centre, with ROTEM (EXTEM, INTEM, FIBTEM) acquired on admission. Machine learning models were developed to predict 30-day mortality using ROTEM alone, using conventional trauma scores (RTS, NISS, GAP, MGAP, TRISS), and their combination. Model performance was assessed through cross validation using AUROC, AUPRC, and specificity at 90% sensitivity. SHAP was used for explainability.

**Results:**

ROTEM alone predicted 30-day mortality with an AUROC of 0.80, comparable to RTS and NISS (both 0.79), and superior to PT–INR (0.63) and base excess (0.58). When combined with ROTEM, specificity significantly improved across all trauma scores, with the greatest gains observed in RTS (0.23 to 0.62) and NISS (0.36 to 0.69) (all *p* < 0.001). Key predictive ROTEM variables included clotting time, clot firmness time, and fibrinolysis indices. Model performance was notably lower in female patients.

**Conclusions:**

A single admission ROTEM test predicted 30-day all-cause mortality with accuracy comparable to traditional trauma scores and outperformed conventional coagulation markers. Integrating ROTEM into established scores significantly enhanced predictive performance. Viscoelastic data appears to hold prognostic information capable of improving long-term trauma outcome assessments.

**Graphical abstract:**

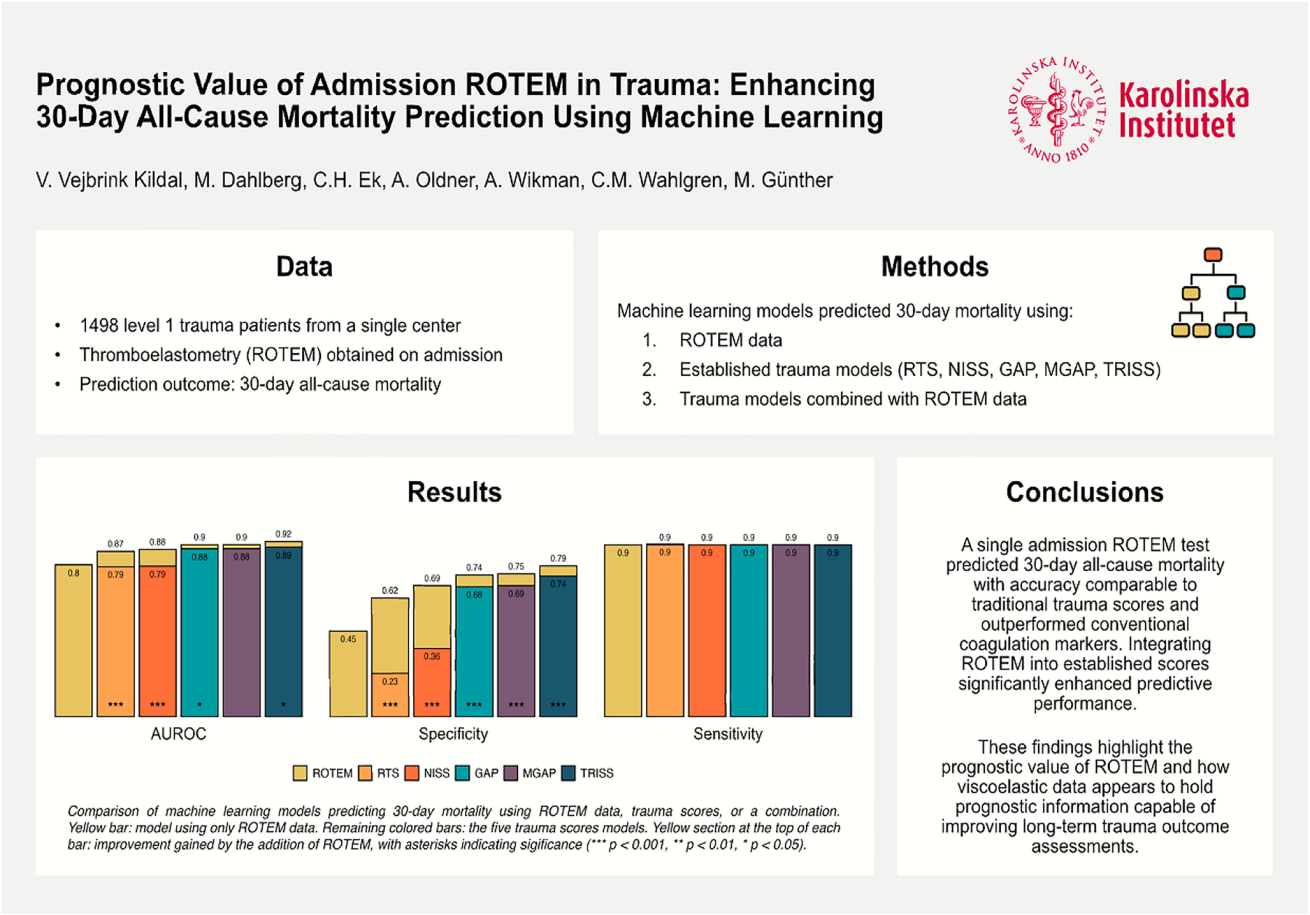

## Background

Trauma remains a leading cause of morbidity and mortality worldwide [1, 2], underscoring the need for precise and reliable outcome prediction models for triage, hospital planning, quality assurance, research, and communicating prognosis to next of kin. Established models, such as the Revised Trauma Score (RTS) [3] and the Trauma and Injury Severity Score (TRISS) [4], were designed to balance predictive power with clinical ease of use. Their reliance on limited data ensures straightforward clinical integration but may restrict predictive accuracy. Furthermore, the emphasis on sensitivity to capture high-risk cases reduces specificity, leading to over-triage and false-positive predictions [5, 6], which limits clinical usefulness. In contrast, modern machine learning models within electronic health records can process complex data in real time and may outperform established clinical prediction models [7]. Despite their potential, such models have not seen widespread adoption in trauma care. To improve future predictive trauma models, clinical variables not yet explored in machine learning contexts should be investigated. Given that exsanguination is the second leading cause of trauma death [8], we hypothesized that early viscoelastic coagulation parameters could serve as a proxy for injury severity, providing insights into both short- and long-term mortality risk.

Rotational thromboelastometry (ROTEM) is a readily available coagulation test that can be performed point-of-care. It measures the physical properties of clot formation by detecting changes in the blood’s viscosity as it clots under low-frequency rotational stress. The output is a time-based curve showing the speed of clot initiation, the rate of clot strengthening, the maximum firmness achieved, and the degree of clot breakdown (lysis). Coagulopathy is a major cause of trauma-related death [9], and conventional coagulation tests, such as Prothrombin Time–International Normalized Ratio (PT–INR), have been shown to predict mortality in trauma patients, despite only reflecting a limited part of the coagulation cascade [10–12]. Compared to conventional tests, specific ROTEM assays target different coagulation pathways, thus providing a more comprehensive assessment of a patient’s haemostatic function in a single analysis. ROTEM can detect coagulopathy and predict the need for massive transfusion earlier than conventional tests and can thus guide trauma resuscitation [13]. The use of ROTEM panels to guide treatment in haemorrhaging trauma patients has been associated with reduced mortality [14]. However, its value for predicting survival remains uncertain. While some studies have linked individual ROTEM parameters to increased mortality risk [15, 16], few have used ROTEM to build models capable of predicting outcomes for individual patients. Where ROTEM has been applied in predictive modelling, the focus has been on short-term coagulation-related death, with analyses typically using simple linear modelling approaches such as logistic regression [17]. 

Given our previous findings that principal component analysis can simplify complex ROTEM data while revealing distinct coagulation profiles [18], we hypothesized that more advanced analytical methods of full ROTEM profiles could extract additional prognostic insights. Specifically, we aimed to evaluate the utility of ROTEM as an independent predictor of 30-day all-cause mortality in trauma patients using advanced non-linear machine learning methods, and to assess whether incorporating ROTEM into established trauma models can improve predictive accuracy by reducing false positive rates. We also examined the contribution of individual ROTEM variables and conducted sex-stratified analyses due to known sex-based differences in ROTEM profiles [19].

## Methods

### Dataset

**Fig. 1:**
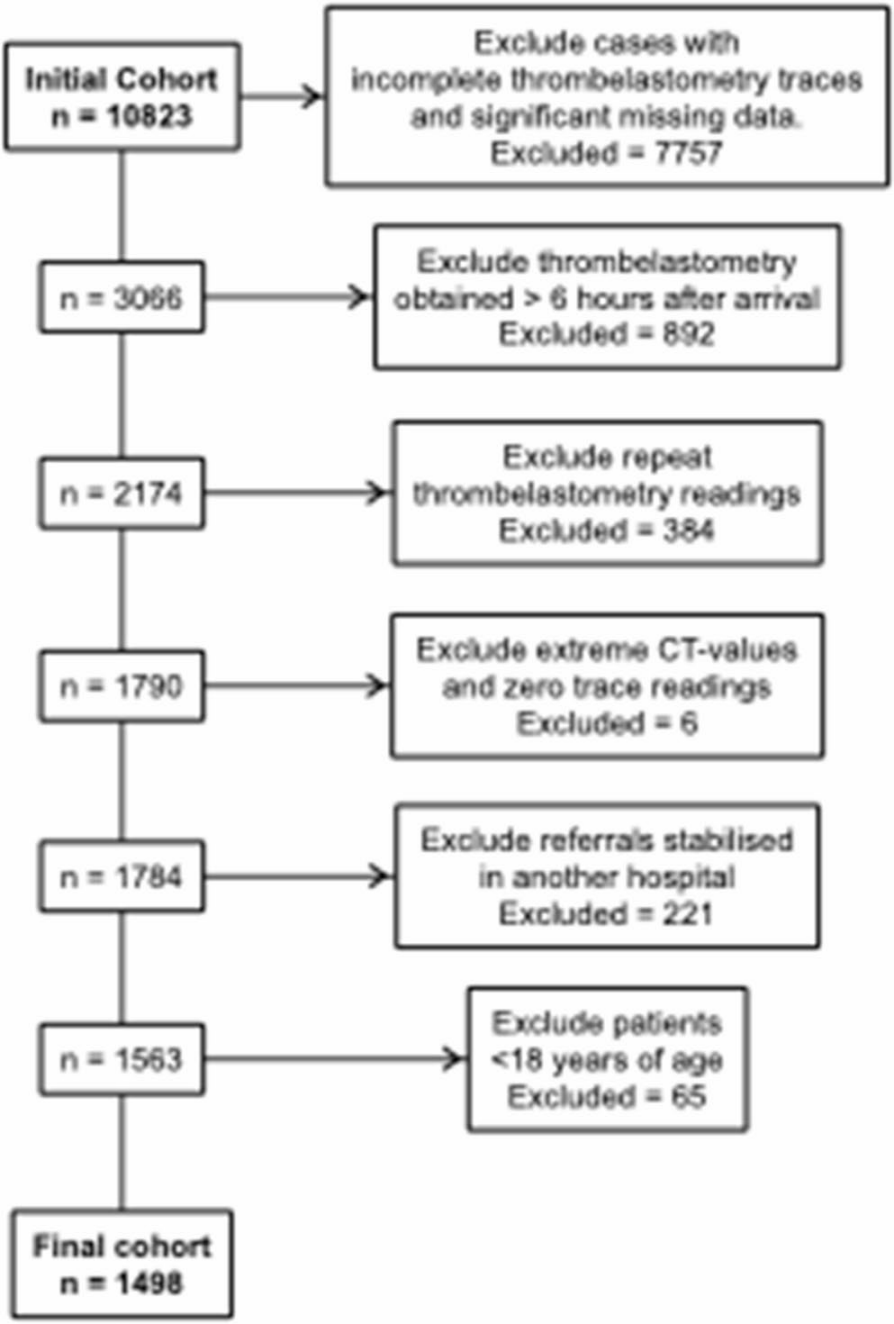
Flowchart detailing the process of patient exclusion and inclusion

The study was approved by the Swedish Ethical Review Authority (Dnr 2020 − 01474). A single-centre, retrospective prognostic cohort study following TRIPOD-AI guidelines (Type 1b) was conducted on patients admitted to a level 1 trauma centre at Karolinska University Hospital in Stockholm, Sweden, between 2015 and 2022. The dataset included all trauma patients aged 18 years or older who had a complete ROTEM test performed within 6 h of admission (ordered based on clinical concern), enrolling patients using consecutive sampling. If several ROTEM tests were performed during this time, only the first was used. Of the 10,823 ROTEM tests acquired during this period, 1,498 patients with unique ROTEM tests were included in the analysis (see Fig. 1 for details on the exclusion process).

Clinical data were extracted from the National Trauma Registry in Sweden (SweTrau), and ROTEM data were extracted from hospital records. The primary outcome was 30-day mortality. Clinical data included sex, age, trauma mechanism, the first vital sign parameters obtained upon hospital arrival (respiratory rate, systolic blood pressure, Glasgow Coma Scale), Injury Severity Score (ISS) [20] and New Injury Severity Score (NISS) [21]. Using this data, the following trauma outcome prediction scores were calculated: the Revised Trauma Score (RTS) [3]the Glasgow coma scale, age, and arterial pressure score (GAP) [22]the mechanism of injury, Glasgow coma scale, age, and arterial pressure score (MGAP) [23]and the Trauma Score and Injury Severity Score (TRISS).

All ROTEM^®^ analyses were performed using the ROTEM^®^ Delta system (Werfen) with liquid reagents for INTEM, EXTEM, and FIBTEM assays (lyophilized (freeze-dried) controls were run weekly). These included the variables clotting time (CT), clot formation time (CFT), maximum clot firmness (MCF), maximum clot firmness time (MCF T), the alpha angle, maximum lysis (ML), lysis index at 30 and 45 min after CT (LI30, LI45), and the amplitude (A) and area under the curve (AR) at five-minute increments (A5 and AR5, A10 and AR10, etc.) for each subgroup.

### Data Preprocessing

In some cases, clinical vital parameter data was missing. Here, the prehospital values obtained in the ambulance prior to hospital arrival were used instead. In cases where prehospital data also was missing but the corresponding RTS score was present, mid-range values within each RTS category were imputed. After this, respiratory rate had 3,9% and GCS 4,5% missing data. ROTEM variables with more than 10% missing data were excluded (INTEM and EXTEM AUC at 15, 20, 25, and 30 min, and FIBTEM AUC at 25 and 30 min). Some ROTEM variables still had missing data (less than 1%: CFT Intem, Alpha Intem, CFT Extem, Alpha Extem, AR15 Fibtem; less than 5%: AR10 Intem, AR10 Extem, AR20 Fibtem). Multiple Imputation by Chained Equations (MICE) was used to impute the missing data based on available variables. All numerical variables were centred, scaled, and log transformed. Outliers were not removed or altered.

The dataset was highly imbalanced, with survivors comprising 92.7% of the sample. This class imbalance can substantially impair classifier performance by introducing bias during training. To circumvent this, we generated balanced training folds by under-sampling the majority class. Model evaluation was performed on the original, non-undersampled test sets, ensuring that the performance metrics were derived from data accurately reflecting the true distribution of outcomes in the population.

### Outcome metrics

The primary outcome metric for model evaluation was the area under the receiver operating characteristic curve (AUROC), which assesses overall model discrimination between survivors and non-survivors. The area under the precision-recall curve (AUPRC), focusing more on the minority class (non-survivors), was also used due to its relevance in imbalanced datasets. To allow for model comparisons using more clinically interpretable metrics, the decision threshold for all models was set corresponding to a sensitivity (recall) of 90% (i.e. a threshold where 90% of non-survivors were correctly identified). At this threshold, additional metrics—specificity, balanced accuracy, and precision—were calculated.

### Machine learning classifier selection

Using the described training and test folds, we applied a variety of machine learning methods to develop a predictive model using the ROTEM variables: logistic regression (glmnet), random forest (ranger), eXtreme Gradient Boosting (XGBoost), k-nearest neighbours (kknn), and support vector machines with a radial basis function kernel (SVM; kernlab). Using default parameters, the logistic regression, random forest, and SVM models achieved similar AUROC scores (0.79, 0.79, 0.80), while the logistic regression and random forest models had the highest AUPRC scores (0.33, 0.28, compared to 0.24 for SVM). When combining ROTEM data with trauma outcome prediction models, the random forest model improved, while the performance of the logistic regression models degraded. Random forest models were thus chosen for all subsequent analyses.

### Hyperparameter tuning and feature selection

The hyperparameters of the model using only ROTEM data were tuned using grid search and cross-validation, optimizing for AUROC. Hyperparameter tuning involves systematically testing different values for parameters to find the combination that yields the best performance. The number of variables considered at each split (mtry) was set to 2 and the minimum number of observations required in a node (min_n) to 7. These settings were applied to all models. Feature selection was conducted by calculating variable importance scores using the ROTEM model to simplify the algorithm and further reduce overfitting risk. Briefly, variable importance quantifies each feature’s contribution to the model’s predictive performance, retaining only the most relevant features. Of the 48 ROTEM variables analysed, the 15 variables with the highest average importance scores were used. After tuning, the AUROC of the ROTEM model improved only marginally, from 0.79 to 0.80, reflecting the model’s stability.

### Model construction and validation

Three machine learning model types were constructed: (1) using ROTEM data alone, (2) using individual trauma outcome prediction models (RTS, NISS, GAP, MGAP, TRISS), and (3) combining ROTEM data with trauma models. The trauma outcome prediction models were incorporated by using their final composite scores as standalone predictors. Since five trauma scoring models were evaluated, this resulted in a total of 11 models (one ROTEM model, five trauma models, and five combined models). Five-fold cross-validation with 10 repeats was chosen over a traditional train-test split. This approach systematically rotates through data subsets, reducing the bias associated with a specific train-test split and providing more reliable performance metrics, particularly for small datasets. The cross-validation produced 50 sets of outcome metrics per model, from which mean, standard deviations, and 95% confidence intervals were calculated.

### Model explainability

SHapley Additive exPlanations (SHAP) values were used to assess the impact of the 15 most predictive variables on model output. SHAP beeswarm plots were generated to visualize the distribution of SHAP values across features, ranked by their median impact, allowing for determination of the impact of separate feature values on prediction outcome.

### Sex-stratified analysis

Model accuracy based on biological sex was evaluated by fitting the model using ROTEM data separately for the male and female subpopulations, with outcome metrics calculated for each group to assess potential differences in predictive accuracy.

### Statistical analysis

The non-parametric DeLong’s test, a standard method for assessing AUROC differences [24], was used to compare AUROC scores between models while accounting for correlation between paired curves [18]. Permutation tests assessed differences in AUPRC, specificity, balanced accuracy, and precision between baseline and combined trauma score models. Metrics from each cross-validation fold were used to compute the mean difference, with 1,000 permutations performed under the null hypothesis. Group labels were shuffled to simulate the null condition, and one-tailed p-values were calculated as the proportion of permuted differences equal to or more extreme than the observed difference, determining if the combined models significantly outperformed the baseline. To compare differences between demographic and ROTEM variables depending on survival status and sex, the non-parametric Wilcoxon Rank-Sum test was used for continuous variables and Fisher´s Exact Test for factor variables. All data processing, model building, statistical analysis, and data visualization were performed using R Statistical Software (RStudio Version 2024.04.2 + 764) within the Tidyverse and Tidymodels ecosystems [25, 26]. 

## Results

### Patient characteristics

Of the 1,498 included patients, 21% were female (*n* = 316) and 7.3% (*n* = 110) died within 30 days. All ROTEM tests were taken within 6 h and 90% within 3.55 h. Non-survivors were, on average, older than survivors, with a median age of 74 compared to 41 years and were more likely to have a higher ASA classification (58% vs. 17% with ASA ≥ 3). They had lower median GCS scores (6 vs. 15) and lower respiratory rates (18 vs. 19) upon hospital arrival. Additionally, non-survivors had lower TRISS, GAP, MGAP, and RTS scores, and higher NISS and ISS scores, reflecting more extensive injuries. Non-survivors had higher PT–INR levels (1.1 vs. 1.0) and lower base excess (−3.5 vs. −0.2). Non-survivors were more likely to be female (31% vs. 20%), have head (85% vs. 42%), face (63% vs. 42%), thorax (51% vs. 37%), spine (35% vs. 22%), isolated upper extremity (57% vs. 47%), and isolated head injuries (18% vs. 12%). Conversely, survivors were more likely to have penetrating injuries (27% vs. 5%) and isolated extremity injuries (11% vs. 1%). Full details provided in Table 1. A stability analysis was performed on the excluded patients, where 30-day mortality was 6%, median ISS was 8 (IQR 1–14), and NISS was 9 (3–21).

**Table 1:** Demographic, clinical, and physiological data at hospital admission for all patients stratified by survival status (30-day mortality). Data are presented as median (interquartile range) or percentages

Demographic, Clinical, and Physiological Data Comparisons					
Variable	All Patients	Survivors	Non-Survivors	P-Value	Significance
Number of Patients	1,498	1,388	110	-	-
Sex: Female	21%	20%	31%	0.009	**
Age	43 (27–61)	41 (27–58)	74 (61–84)	< 0.001	***
ASA 1–2	80%	83%	42%	< 0.001	***
ASA ≥ 3	20%	17%	58%	< 0.001	***
Injury Mechanism: Penetrating	26%	27%	5%	< 0.001	***
Systolic Blood Pressure	135 (120–150)	135 (120–150)	130 (100–160)	0.372	NS
Respiratory Rate	19 (16–22)	19 (16–22)	18 (10–20)	0.005	**
Glasgow Coma Scale	15 (13–15)	15 (13–15)	6 (3-12.8)	< 0.001	***
PT–INR	1 (1-1.1)	1.0 (1-1.1)	1.1 (1-1.3)	< 0.001	***
Base Excess	−0.3 (−3.5−1.9)	−0.2 (−3.1−2)	−3.5 (−9.6−0.6)	< 0.001	***
RTS Final Score	7.84 (7-7.8)	7.84 (7.6–7.8)	5.44 (4.1–6.9)	< 0.001	***
ISS Score	10 (5–21)	10 (4–18)	26 (17.2–32.2)	< 0.001	***
NISS Score	14 (5–27)	13.0 (4.8–27)	36.5 (26.2–50)	< 0.001	***
GAP Score	22 (19–24)	22 (20–24)	12 (10-17.8)	< 0.001	***
MGAP Score	25 (22–27)	25 (23–28)	16 (13–21)	< 0.001	***
TRISS Score	0.99 (1–1)	0.993 (1–1)	0.559 (0.2–0.9)	< 0.001	***
Region 1 Head	45%	42%	85%	< 0.001	***
Region 2 Face	43%	42%	63%	< 0.001	***
Region 3 Neck	9%	9%	6%	0.482	NS
Region 4 Thorax	38%	37%	51%	0.004	**
Region 5 Abdomen	23%	23%	21%	0.639	NS
Region 6 Spine	23%	22%	35%	0.003	**
Region 7 Upper Extr	48%	47%	57%	0.048	*
Region 8 Lower Extr	49%	49%	52%	0.553	NS
Region 9 External And Other	4%	4%	4%	1	NS
Isolated Extremity Trauma	10%	11%	1%	< 0.001	***
Isolated Head Trauma	12%	12%	18%	0.049	*

*P*-values compare the survivor and non-survivor groups. *** *p* < 0.001, ** *p*< 0.01, * *p* < 0.05, NS non-significant

### Comparing ROTEM variables based on survival status

Several ROTEM variables displayed significant differences between survivors versus non-survivors (Table 2). Non-survivors had significantly lower ML Intem, MCF T Intem, A20 Extem, A20 Intem, while LI45 Intem, CT Extem, LI30 Intem, CT Fibtem, MCF T Fibtem were higher.

**Table 2:** Comparisons of the top 15 most predictive ROTEM variables stratified by survival status (30-day mortality). Data are presented as median (interquartile range)

ROTEM Data Comparisons				
Stratified by Survival Status				
Variable	Survivors	Non-Survivors	*P*-Value	Significance
LI45 INTEM	97 (95–98)	98 (96–100)	< 0.001	***
ML FIBTEM	1 (0–4)	0 (0–3)	0.092	NS
ML INTEM	6 (4–9)	4 (2–7)	< 0.001	***
LI45 EXTEM	98 (96–99)	98 (96–99)	0.214	NS
ML EXTEM	6 (4–8)	5 (3–9)	0.159	NS
CT EXTEM	61 (56–68)	71 (62-98.8)	< 0.001	***
LI30 INTEM	100 (99–100)	100 (100–100)	0.021	*
CT FIBTEM	59 (54–66)	69 (60.2–92)	< 0.001	***
LI45 FIBTEM	100 (98–100)	100 (100–100)	0.109	NS
MCF T INTEM	1466.0 (1293.8–1672)	1647.5 (1380.5-1888.5)	< 0.001	***
MCF T FIBTEM fFFIBTEM	1123.5 (896.5-1365.2)	1306.0 (888.8–1695)	0.002	**
A20 EXTEM	61.0 (57–64)	58.5 (54–64)	0.006	**
A20 INTEM	60 (56–63)	58 (53–63)	0.037	*
MCF T EXTEM	1620.5 (1451-1800.2)	1649.0 (1426.2-1948.2)	0.491	NS
LI30 EXTEM	100 (100–100)	100 (100–100)	0.046	*

*** *p* < 0.001, ** *p* < 0.01, * *p* < 0.05, NS non-significant

### Model Performance: Comparing the model using only ROTEM data to the trauma models

**Fig. 2:**
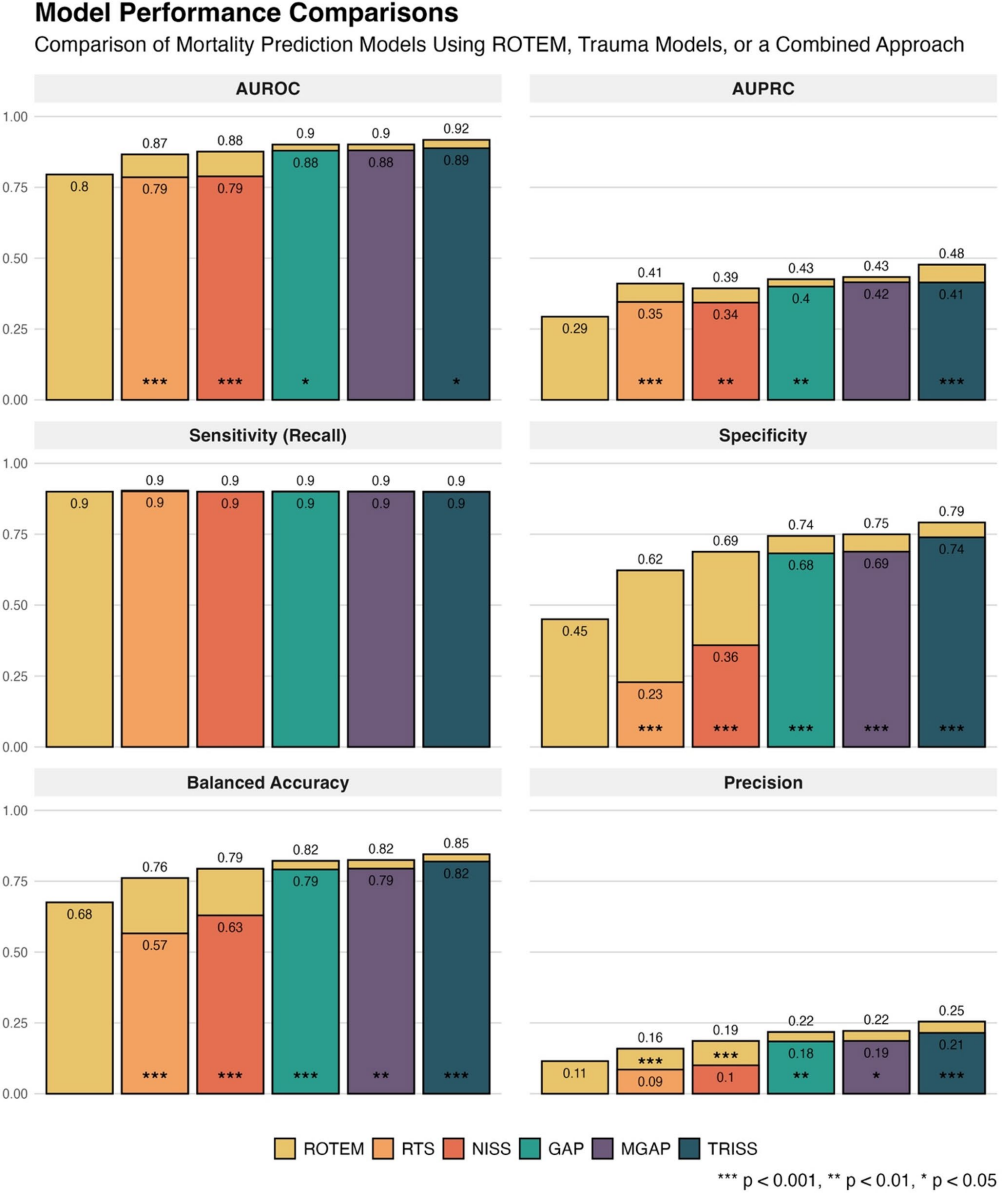
Comparison of the performance of mortality prediction models using either ROTEM data, trauma scores, or a combination of both. The yellow bar demonstrates the performance of the model using only ROTEM data, while the remaining bars represent models based on five trauma scores (RTS, NISS, GAP, MGAP, and TRISS). The yellow section at the top of each bar indicates the improvement gained by adding ROTEM data to the trauma score models. The values inside each bar show the performance of the individual models, whereas the numbers above each bar display the results when ROTEM data was combined with trauma scores. The threshold for each model was set for a sensitivity (recall) of 90%, allowing for comparisons of specificity, balanced accuracy, and precision. The asterisks indicate if the difference between the trauma model and the combined model was significant. *** *p* < 0.001, ** *p* < 0.01, * *p* < 0.05

**Fig. 3:**
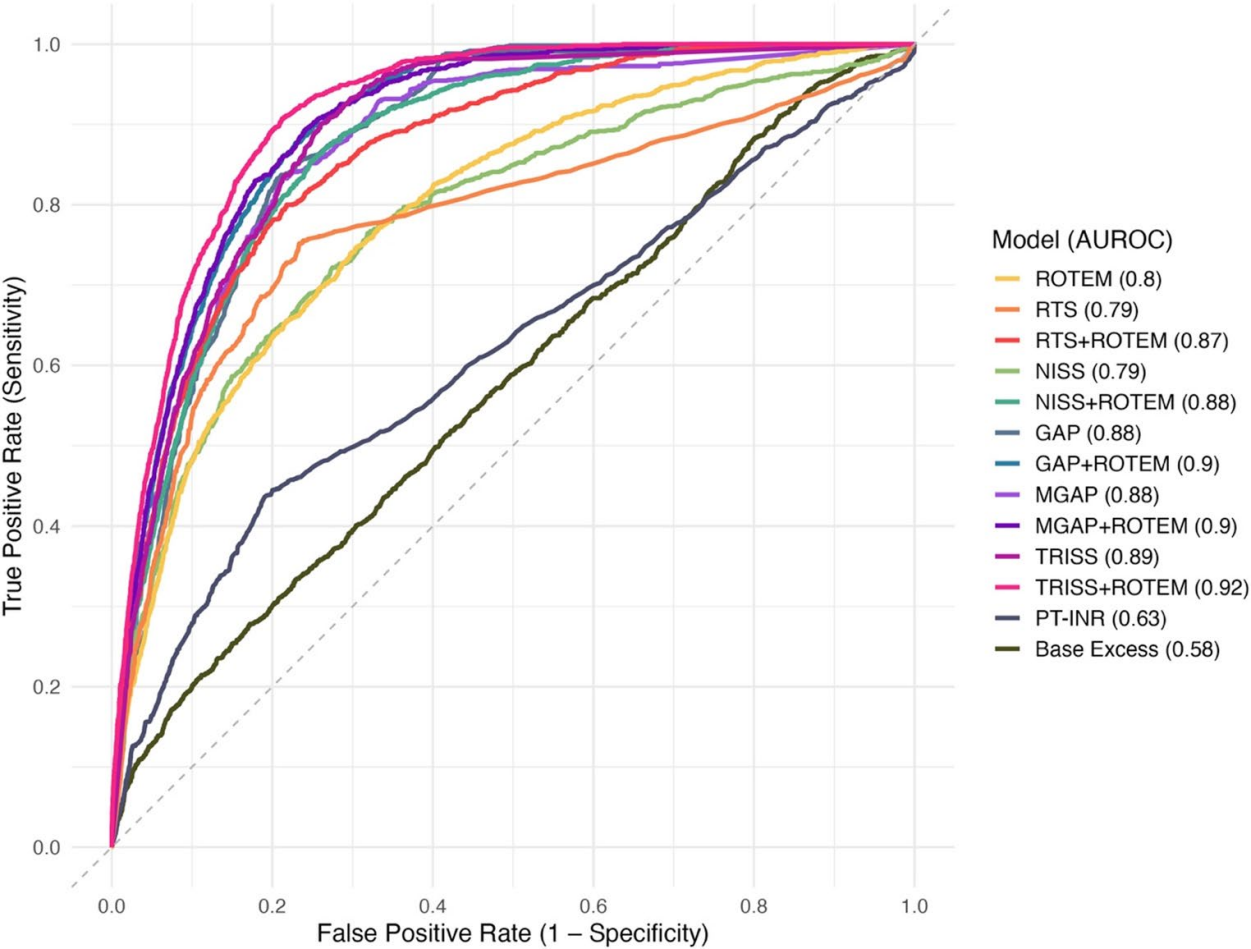
Receiver Operating Characteristic (ROC) curves for all models predicting 30-day mortality. Each curve shows the trade-off between sensitivity and 1 – specificity across thresholds. Area under the ROC curve (AUROC) values are shown in the legend. Models using ROTEM variables are colour-paired with their corresponding trauma score baseline. PT–INR and base excess models are shown as separate reference predictors

The model using only ROTEM data independently predicted 30-day mortality with an AUROC of 0.795 (95% Confidence Interval 0.781–0.810), compared to the RTS model, with AUROC 0.786 (0.773–0.799; *p* = 0.323 when comparing with the ROTEM model), NISS with 0.789 (0.772–0.806, *p* = 0.715), GAP with 0.880 (0.871–0.888, *p* < 0.01), MGAP with 0.880 (0.869–0.892, *p* < 0.001), and TRISS with 0.888 (0.881–0.896, *p* < 0.001). Complementary metrics – AUPRC, specificity, balanced accuracy, and precision – were also calculated. At a sensitivity (recall) of 90%, the ROTEM model demonstrated significantly better specificity and balanced accuracy compared to both the RTS and NISS models (*p* < 0.001). Precision was significantly better for ROTEM compared to NISS (*p* < 0.001) but not RTS (*p* = 0.129). See Figs. 2 and 3 for full model comparisons. Separate models were developed using PT–INR and base excess as sole predictors. PT–INR achieved an AUROC of 0.629 (95% CI: 0.612–0.646), an AUPRC of 0.170 (0.153–0.188), and a specificity of 0.163 (0.131–0.195) at 90% sensitivity. Base excess achieved an AUROC of 0.578 (0.561–0.596), an AUPRC of 0.133 (0.115–0.150), and a specificity of 0.173 (0.157–0.189) at the same sensitivity threshold. AUROC curves are presented in Fig. 3.

### Model Performance: Comparing the standalone trauma models to the combined models

To evaluate the cumulative impact on prediction accuracy, ROTEM data were added to the models constructed using only trauma scoring systems, improving the AUROC by 0.048 ± 0.033 on average. As seen in Fig. 2, RTS improved from 0.786 (0.773–0.799) to 0.866 (0.857–0.876) (*p* < 0.001), NISS from 0.789 (0.772–0.806) to 0.876 (0.868–0.885) (*p* < 0.001), GAP from 0.880 (0.871–0.888) to 0.901 (0.896–0.907) (*p* < 0.05), MGAP from 0.880 (0.869–0.892) to 0.902 (0.894–0.909) (*p* = 0.078), and TRISS from 0.888 (0.881–0.896) to 0.918 (0.912–0.923) (*p* < 0.05). All additional metrics demonstrated significant improvements across all models except for the MGAP AUPRC improvement. The most notable gains in AUPRC were observed for RTS, NISS and TRISS. At a cutoff yielding 90% sensitivity, ROTEM improved the specificity for RTS from 0.228 (0.175–0.282) to 0.622 (0.612–0.633), NISS from 0.359 (0.331–0.387) to 0.688 (0.678–0.698), GAP from 0.682 (0.656–0.709) to 0.744 (0.738–0.749), MGAP from 0.688 (0.681–0.696) to 0.750 (0.744–0.755), and TRISS from 0.739 (0.730–0.748) to 0.791 (0.785–0.798) (all *p* < 0.001). For balanced accuracy and precision, large gains were seen for RTS and NISS, with smaller improvements for GAP, MGAP, and TRISS.

### The most predictive variables

**Fig. 4:**
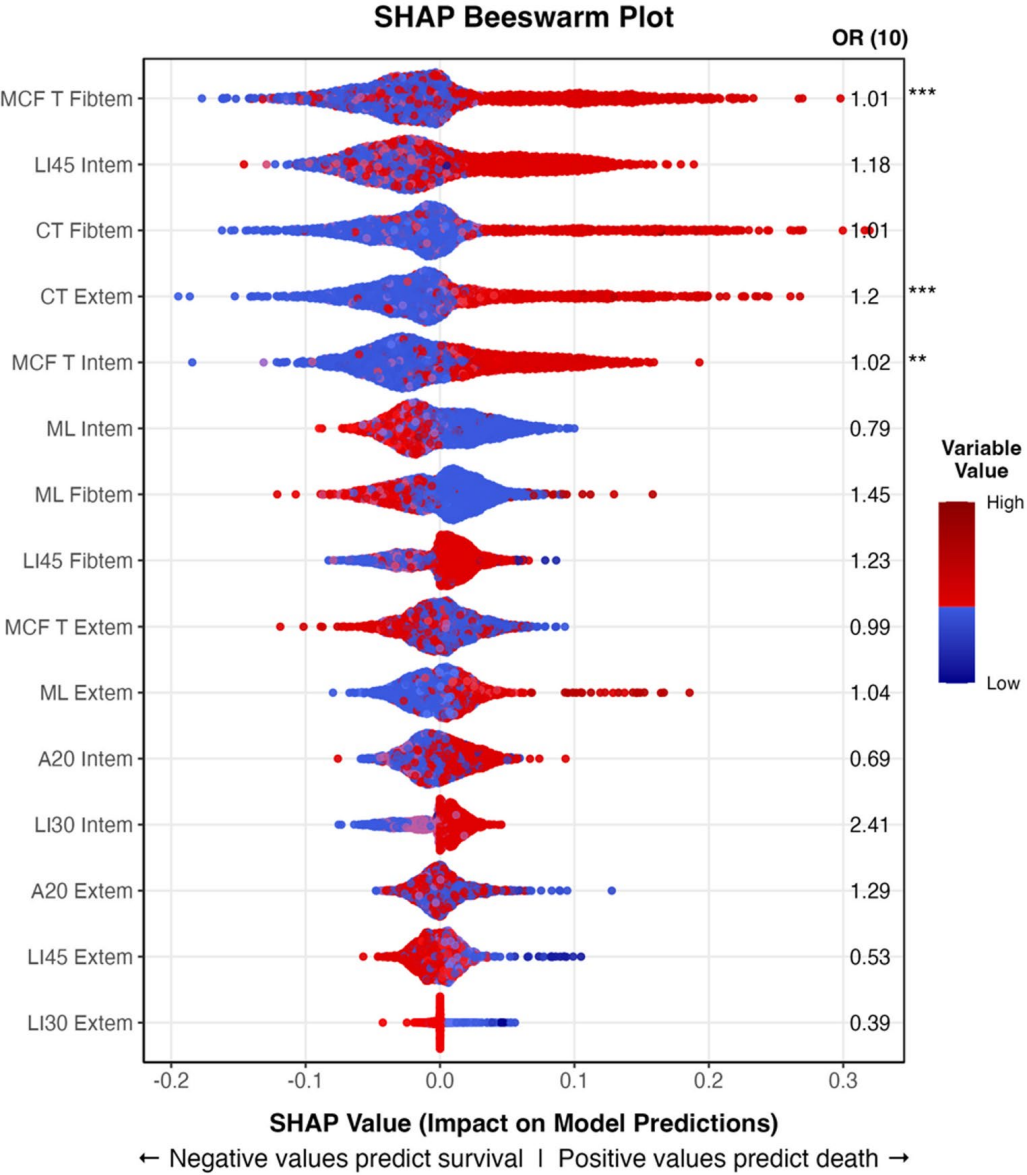
SHAP beeswarm plot with odds ratios. SHAP plots provide a visual explanation of feature influence on model predictions by showing how much each feature pushes the prediction towards the different outcomes. The x-axis represents SHAP values, with each point corresponding to a single observation in the dataset. The colours represent the original (scaled) value of the datapoint (red indicating above median and blue indicating below median values). The position of the point along the x-axis reflects how much that specific feature influences the prediction for that data point, either toward survival (negative SHAP values, to the left) or death (positive SHAP values, to the right). Features are displayed in descending order of importance, based on the mean absolute SHAP value, which quantifies each feature’s overall contribution to model output (wider spreads indicate greater importance). Odds Ratios for a 10 unit increase in the ROTEM variable (OR 10), shown to the right of each feature, are derived from a separate logistic regression model and were calculated as a supplementary measure to provide further context to the SHAP findings. An OR greater than 1 indicates an increased likelihood of death with a 10 unit increase in the variable, while an OR less than 1 suggests a lower likelihood of death. For example, the OR of 1.2 for CT Extem means that for every 10-unit increase, the odds of death increases by 20% - supported by the red (high) SHAP values to the right in the plot, indicating that the model predicts that high numbers of CT Extem lead to higher risk of death. Asterisks indicate the statistical significance of the odds ratios from the logistic regression model (*** *p* < 0.001, ** *p* < 0.01, * *p* < 0.05)

Of all 48 ROTEM variables analysed, the 15 strongest predictors that were included in the models are shown in the SHAP plot in Fig. 4. The three most influential ROTEM variables for predicting 30-day mortality was MCF T Fibtem, LI45 Intem, and CT Fibtem, with higher values shifting the predictions toward non-survival.

### Bias analysis

Sex-stratified analysis revealed a significantly lower predictive performance in female compared to male patients with an AUROC of 0.588 (95% CI: 0.556–0.620) versus 0.841 (CI 0.829–0.853) (*p* < 0.001), and AUPRC 0.199 (CI 0.170–0.228) versus 0.336 (CI 0.304–0.368) (*p* < 0.001). Mortality was higher in female patients (10.8%) compared to male patients (6.4%). Significant ROTEM differences between sexes were found in both the survivor and non-survivor groups, presented in Table 3. Age-stratified analysis revealed that the model performed better in younger patients < 65 years (AUROC 0.805 (0.782–0.827), AUPRC 0.242 (0.196–0.288)) than in older patients (AUROC 0.643 (0.623–0.662), AUPRC 0.383 (0.347–0.418)). Notably, mortality was substantially lower in younger patients (2.8%) compared to older patients (25.2%), which explains the higher AUPRC in the older group, as higher baseline prevalence increases the achievable precision–recall performance.

**Table 3:** Comparisons of the top 15 most predictive ROTEM variables stratified by sex and survival status (30-day mortality). Data are presented as median (interquartile range)

ROTEM Data Comparisons								
Stratified by Sex and Survival Status								
Variable	Female Survivors	Male Survivors	P-Value	Significance	Female Non-Survivors	Male Non-Survivors	*P*-Value	Significance
LI45 INTEM	97 (95–99)	97 (95–98)	0.159	NS	98 (95–99)	99 (96–100)	0.078	NS
ML FIBTEM	0 (0–3)	1 (0–4)	< 0.001	***	0 (0–2)	0 (0–3)	0.77	NS
ML INTEM	6.0 (4–8)	6.0 (4–9)	0.099	NS	5.5 (3-8.8)	4.0 (2–7)	0.128	NS
LI45 EXTEM	98 (96–99)	98 (96–99)	0.484	NS	97 (94.2–99)	98 (96–99)	0.279	NS
ML EXTEM	6 (4–8)	6 (4–9)	0.251	NS	6 (3-10.8)	5 (2-8.2)	0.255	NS
CT EXTEM	59.0 (54–66)	62.0 (57–69)	< 0.001	***	63.0 (58–71)	77.5 (68-102.2)	< 0.001	***
LI30 INTEM	100 (99–100)	100 (99–100)	0.817	NS	100 (99.2–100)	100 (100–100)	0.264	NS
CT FIBTEM	57.0 (52–62)	60.0 (55–66)	< 0.001	***	63.5 (57.2–70)	75.5 (62-101.2)	0.003	**
LI45 FIBTEM	100 (99–100)	100 (98–100)	< 0.001	***	100 (99–100)	100 (100–100)	0.529	NS
MCF T INTEM	1456.5 (1270.5-1673.5)	1471.0 (1299–1671)	0.539	NS	1539.0 (1279.2–1789)	1667.0 (1432.2-1950.2)	0.075	NS
MCF T FIBTEM	1150.5 (917.2-1452.5)	1120.5 (889.2–1349)	0.061	NS	1108.5 (879–1492)	1330.5 (927.2–1724)	0.112	NS
A20 EXTEM	63 (58–66)	60 (57–64)	< 0.001	***	62 (55.5–65)	58 (54–63)	0.152	NS
A20 INTEM	62.0 (57–65)	59.0 (56–62)	< 0.001	***	62.5 (55.2–64.8)	57.0 (53–62)	0.044	*
MCF T EXTEM	1610.5 (1441.8-1790.5)	1625.5 (1453.2-1802.5)	0.397	NS	1550.0 (1297.5-1910.5)	1662.5 (1449.8-1955.5)	0.186	NS
LI30 EXTEM	100 (100–100)	100 (100–100)	0.373	NS	100 (100–100)	100 (100–100)	0.593	NS

*** *p* < 0.001, ** *p* < 0.01, * *p* < 0.05, NS non-significant

## Discussion

In this study, we demonstrate that a single ROTEM test performed on admission in trauma patients can independently predict 30-day all-cause mortality with accuracy comparable to that of established trauma outcome prediction models. The ROTEM-based model achieved an AUROC of 0.80, performing similarly to the RTS and NISS scores (both 0.79), and outperforming other coagulation markers (PT–INR, AUROC 0.63). Incorporating ROTEM data into established trauma models (RTS, NISS, GAP, MGAP, and TRISS) significantly enhanced their predictive performance, reducing false positive predictions across all models. Importantly, our aim was not to develop a new trauma prediction tool, but to evaluate whether ROTEM adds prognostic insight into the role of coagulation in all-cause trauma mortality. By utilising full ROTEM panels and advanced predictive models in one of the larger ROTEM trauma datasets to date, we show that ROTEM holds prognostic information capable of predicting long-term outcomes for individual trauma patients.

A variety of trauma outcome prediction models are currently used, with some focusing on the anatomical description of injuries and others on physiological parameters. The most widely used models combine both anatomical and physiological data. The challenge of integrating diverse factors such as pre-existing conditions, age, immunological differences, and genetic predispositions, has made the development of a universally applicable trauma scoring system both complex and, perhaps, unattainable. An effective trauma scoring system must meet key criteria, including accuracy, specificity, and reliability. When these criteria are fulfilled, the system can serve several important functions, including predicting trauma outcomes such as mortality. Additionally, it can be used as a triage tool, to support quality improvement and injury prevention programs, and provide a robust framework for trauma research [27]. However, while maximizing sensitivity to avoid missing high-risk patients, many trauma models suffer from high false-positive rates, where many survivors are incorrectly predicted as non-survivors [5, 6]. The high false positive rate limits the accuracy of current models for quality assurance and research, and, if used for clinical decision making, might lead to alarm fatigue and limit their clinical usefulness. In contrast, machine learning models integrated into electronic health records can be designed to leverage a broader range of patient data to improve trauma predictions and reduce false positives [7]. 

This study has shown that machine learning analysis of ROTEM can extract prognostic insights from coagulation dynamics, perhaps by detecting coagulopathies that traditional trauma models may overlook, while also serving as a proxy for initial injury severity. The addition of ROTEM led to significant increases in predictive performance in the models with initially lower predictive accuracy–RTS and NISS–with AUROC rising from 0.79 to 0.87 and from 0.79 to 0.88, rivalling the accuracy of the best performing models. Significant, although less pronounced, improvements were seen in the models with higher baseline accuracy (TRISS and GAP). The low specificity and precision scores resulting from the chosen 90% sensitivity threshold highlight a significant limitation of current trauma models: a concerning rate of false positives, where only 9–21% of non-survival predictions were correct. Adding ROTEM data significantly improved both specificity and precision in all models, where the large increases in specificity for RTS and NISS are particularly noteworthy. Initially, only 23% and 36% of survivors were correctly classified by these models. When adding ROTEM, specificities increased to 62% and 69%, significantly enhancing model accuracy. Along with the significant specificity improvements also seen in the higher-performing models GAP, MGAP, and TRISS (specificities of 0.68, 0.69, and 0.74 improved to 0.74, 0.75, and 0.79), these findings highlight the potential clinical impact of incorporating viscoelastic data into existing trauma prediction models. The dataset had a, for trauma datasets, typical class imbalance, with significantly more survivors than non-survivors. Such imbalance makes it challenging to develop models that accurately predict the minority class (non-survivors) and to reliably evaluate their performance. While AUROC is an important metric, it can be misleading in this context, as it may appear high due to correct classification of the majority class even if the model struggles with the minority class. The observed increase in AUPRC—derived from precision and recall—better demonstrated improved ability to correctly classify non-survivors. Given the expected baseline AUPRC of 0.073 (reflecting 7.3% non-survivors), all models exceeded this substantially, achieving scores above 0.29. Notably, the largest AUPRC improvement with ROTEM was seen in the best-performing model, TRISS, increasing from 0.41 to 0.48, demonstrating that meaningful improvements in identifying non-survivors were achievable also in the best performing models.

The AUROC of 0.80 in our ROTEM-only model can be compared to a previous study using thromboelastography (TEG) to predict coagulation-related mortality in trauma, which reported an impressive AUROC of 0.89 [17]. The difference can be explained by their focus on solely predicting deaths specifically due to coagulopathy, defined as patients who died after receiving 10 or more blood units. In contrast, we predicted long-term (30-day) all-cause mortality, a broader outcome that naturally lowers achievable AUROC scores. Thus, our results underscore the potential of ROTEM to improve the prediction of overall trauma outcomes rather than just coagulation-related deaths, likely by conveying information on initial trauma severity. The model identified variables such as maximum lysis (ML) and lysis index (LI) as top predictors of mortality. Notably, the association between lysis markers (ML and LI) and mortality aligns with excessive fibrinolysis as a known marker of trauma induced coagulopathy and poor survival [28]. Random Forest models are capable of handling non-liner interactions, potentially making them more capable of handling several intricate ROTEM predictor variables modelled together. This is highlighted in Fig. 4, where, for example, high (red) lysis values in ML Fibtem pushed predictions towards survival (to the left in the plot), while very high (dark red) and low (blue) values pushed predictions towards death (to the right). A similar pattern, but in the opposite direction, was seen in LI45 Fibtem, which measures the percentage of remaining clot at 45 min: low values predicted survival, while very low and high values predicted death. A clear correlation was observed between SHAP values and logistic regression odds ratios (Fig. 4), with ROTEM variables with high values associated with mortality having odds ratios above 1. Interestingly, of the top 15 most important variables identified by the random forest model, only three were significantly associated with 30-day mortality when analysed using logistic regression: MCF T Fibtem, CT Extem and MCF T Intem. Logistic regression captures independent, linear relationships with the outcome, potentially underestimating a variable’s broader predictive significance, while multicollinearity between ROTEM variables, which can reduce statistical power and mask the significance of individual predictors in regression models, could also contribute to the observed difference.

PT–INR has previously shown strong predictive properties for trauma mortality (AUROC 0.83) [11] but performed poorly in our cohort (AUROC 0.629, specificity 0.163 at the 90% sensitivity threshold), likely due to smaller differences between survivors and non-survivors (1.0 vs. 1.1 in our study vs. 1.1 vs. 1.4 in the prior study). This makes the performance of ROTEM particularly noteworthy, as it effectively distinguished outcomes despite minimal variation in coagulation profiles between survivors and non-survivors based on PT–INR, suggesting its predictive power could be even greater in populations with more pronounced haemostatic differences.

The ROTEM model performed notably worse in female patients than in males (AUROC 0.59 versus 0.84). There are several possible causes. The discrepancy is likely influenced by the lower female prevalence in the dataset (21%, *n* = 316/1498) and addressing class imbalance through methods such as oversampling the minority class or undersampling the majority class could help mitigate this issue. However, factors beyond class imbalance might also contribute to sex bias, such as anatomical or physiological differences, differing treatment approaches, or inadequate representation of sex-specific features in the model. Of the 316 females, 26 (8.2%) presented with penetrating injuries, compared to 361 (30.5%) of the 1182 males, a disparity that could have a physiological impact on predictive outcomes. Significant sex differences were also seen in ROTEM variables both in survivors and non-survivors (Table 3). Previous studies have shown that ROTEM variables differ depending on sex in trauma patients, and hypercoagulability in females has been hypothesized as a survival advantage [19]. To our knowledge, this is the first study to present sex differences in ROTEM data stratified by survival status, providing deeper insights into how sex may influence ROTEM outcomes. Similarly, we found that the model performed better in younger patients than older, highlighting the need for further research to elucidate underlying factors.

### Limitations and future directions

Several limitations should be noted. First, blood products were administered but the timing of administration was not recorded, preventing us from determining whether transfusions occurred before or after ROTEM sampling and, thus, whether they influenced predictive accuracy. The 6-hour timeframe of ROTEM acquisition might have exacerbated this issue by allowing more time for additional treatment. Next, data on anticoagulant treatment was not available, and could thus be a confounder in the analysis. Information on routine prehospital administration of tranexamic acid was also lacking and could have influenced lysis parameters. Despite this, several lysis measures remained among the top 15 strongest predictors of mortality, showing that the model was still able to extract meaningful predictive information despite potential lysis inhibition. Furthermore, both the risks of overfitting and underfitting must be considered. Overfitting can occur when the model learns patterns too specific to the training data, while underfitting may result from failing to capture key patterns, which can occur for example when undersampling is used during training to balance the dataset. We utilized robust cross validation methods for model evaluation to account for such risks. Nonetheless, validating these findings with external, independent trauma datasets is necessary to confirm their generalizability. The amount of missing data at emergency room arrival was relatively small but reflects current challenges when analysing healthcare data. Imputation was performed only as a last measure to not lose important clinical information and should not have significantly skewed predictions, but any imputation inherently introduces some uncertainty. Selection bias due to the exclusion criteria could have affected results, however, stability analysis, where the excluded patients were compared with the studied sample, showed only small differences in survival and trauma severity between the groups. Next, the final composite scores of the traditional trauma outcome models were used instead of their underlying variables to preserve their unaltered, original, structure, while isolating ROTEM’s contribution. This approach allowed direct comparisons but the roles of individual score components and their interactions with ROTEM may be obscured. Alongside the established prediction models, we used PT-INR and base excess as single-parameter benchmarks; however, we could not evaluate other commonly reported markers—such as lactate or fibrinogen—as they were not available. We did not have data on cause of death and could thus not distinguish patients with traumatic brain injury and massive haemorrhage. Lastly, real-time integration of ROTEM data into machine-learning models is currently impractical, as ROTEM systems often are disconnected from electronic health records. To enable ROTEM-based prediction and decision-making, results must be supplied in a standardized, machine-readable format that can be auto-fed into models.

## Conclusions

This study demonstrated that a single ROTEM coagulation test obtained upon admission in trauma patients can independently predict 30-day mortality, with accuracy comparable to established trauma outcome prediction models. Moreover, ROTEM outperformed other coagulation and lab markers, and incorporating ROTEM data into the RTS, NISS, GAP, MGAP, and TRISS models enhanced their predictive performance and significantly reduced false positive predictions. These findings suggest that viscoelastic data holds prognostic information capable of improving long-term trauma outcome assessments.

## Data Availability

The datasets analyzed during the current study are not publicly available to protect patient confidentiality.

## Abbreviations

ROTEM: Rotational thromboelastometry
GCS: Glasgow Coma Scale
ISS: Injury Severity Score
NISS: New Injury Severity Score
RTS: Revised Trauma Score
GAP: Glasgow coma scale, Age, and arterial Pressure score
MGAP: Mechanism of injury, Glasgow coma scale, Age, and arterial Pressure score
TRISS: Trauma Score and Injury Severity Score
AUPRC: Area Under the Precision-Recall Curve
PT–INR: Prothrombin Time–International Normalized Ratio

## ROTEM variables

CT: clotting time
CFT: clot formation time
MCF: maximum clot firmness
MCF T: maximum clot firmness time
ML: maximum lysis
LI: lysis index (LI30 and LI45: lysis index at 30 and 45 minutes))
A: amplitude at five-minute increments (A5, A10 etc)
AR: area under the curve at five-minute increments (AR5, AR10 etc)
